# Retraction notice to “A study on the strength and durability characteristics of fiber-reinforced recycled aggregate concrete modified with supplementary cementitious material” [HLY **9** (2022) e19978]

**DOI:** 10.1016/j.heliyon.2024.e36697

**Published:** 2024-08-22

**Authors:** Osama Zaid, Fadi Althoey, Rebeca Martínez García, Jesús de Prado-Gil, Saleh Alsulamy, Mohammed Awad Abuhussain

**Affiliations:** a Department of Civil Engineering, Swedish College of Engineering and Technology, 47070, Wah Cantt, Punjab, Pakistan; b Department of Civil Engineering, College of Engineering, Najran University, Najran, Saudi Arabia; c Department of Mining Technology, Topography, and Structures, University of Le'on, Campus of Vegazana S/n, 24071, Le'on, Spain; d Department of Architecture and Planning, College of Engineering, King Khalid University, Abha, 61421, Saudi Arabia; e Architectural Engineering Department, College of Engineering, Najran University, Saudi Arabia

This article has been retracted: please see Elsevier Policy on Article Withdrawal (https://www.elsevier.com/locate/withdrawalpolicy).

This article has been retracted at the request of the Journal Editor.

A concern was raised about the reuse of identical figures in multiple publications by the same group of authors. The journal editor asked the authors to explain and provide raw data and other evidence that the authors felt would be useful. The corresponding author provided raw data, but it was found that the raw data did not match the values presented in the paper for the XRD analysis of wheat straw ash. As a result, the editor has lost confidence in the article and has decided to retract it. The corresponding author disagrees with the retraction of the article and disputes the grounds for it.

We apologize to the readers of the journal for not having identified this issue during the submission process.

